# Shared Information between Residues Is Sufficient to Detect Pairwise Epistasis in a Protein

**DOI:** 10.1371/journal.pgen.1006471

**Published:** 2016-12-22

**Authors:** Aditi Gupta, Christoph Adami

**Affiliations:** 1 Center for Infectious Diseases, New Jersey Medical School, Rutgers University, Newark, New Jersey, United States of America; 2 Department of Microbiology & Molecular Genetics, Michigan State University, East Lansing, Michigan, United States of America; 3 BEACON Center for the Study of Evolution in Action, Michigan State University, East Lansing, Michigan, United States of America; University of Michigan, UNITED STATES

In a comment [1] on our manuscript “Strong Selection Significantly Increases Epistatic Interactions in the Long-Term Evolution of a Protein” [2], Dr. Crona challenges our assertion that shared entropy (that is, information) between two residues implies epistasis between those residues by constructing an explicit example of three loci (say A, B, and C), in which A and B are epistatically linked (leading to shared entropy between A and B) and A and C are also epistatically dependent (leading to shared entropy between A and C) so that loci B and C are correlated (share entropy). She goes on to assert that (as per her examples) even though there will be correlations (and thus shared entropy) between the residues at loci B and C, there is no pairwise epistasis between loci B and C, contradicting our assertion in [1] that shared entropy implies epistasis.

The disagreement is based on two different interpretations of the meaning of pairwise epistasis, and the comment gives us an opportunity to discuss those.

We do not disagree that epistasis refers to mutational effects that are conditional on the states of other alleles. In our paper, we are in particular interested in pairwise epistasis—that is, how the fitness effects of mutations at two loci depend on each other. Of course, the dependence between those two loci could depend on the state of many other alleles in the genome. The different interpretations of epistasis hinge upon whether a quantitative assessment of the epistasis between two loci should be conditional on the state of other loci in the genome, or whether instead we should consider the state of these loci averaged over what their state would be in a population at mutation–selection balance. Crona’s example helps us illustrate that distinction.

A fitness landscape constructed in the supplementary information of [1] (example 1) has three loci: A, B, and C. The values given are (note that in [1], the log-transformed relative fitnesses are given instead):

$$w_{000}=1,w_{001}=0.1,w_{010}=0.1,w_{011}=0.01,w_{100}=1.1,w_{101}=1.1,w_{110}=1.21,w_{111}=1.331$$ (1)

The values are judiciously chosen such that $\frac{w_{000}w_{011}}{w_{010}w_{001}}=\frac{w_{100}w_{111}}{w_{110}w_{101}}=1$.

Crona defines the epistasis between loci B and C as either

$$\varepsilon_{BC}{}^{A=0}=\text{log}\left(\frac{w_{000}w_{011}}{w_{010}w_{001}}\right)$$ (2)

or

$$\varepsilon_{BC}{}^{A=1}=\text{log}\left(\frac{w_{100}w_{111}}{w_{110}w_{101}}\right)$$ (3)

Note that these values are conditional on the state of locus A, but for the case discussed here they happen to be equal and vanishing. Clearly, this is a special case. In general, the pairwise epistasis conditional on the state of another locus can depend on that state, and if there are *n* other binary loci, then there could be in principle 2^*n*^ different values for the pairwise epistasis. Surely this is not satisfactory, as pairwise epistasis then would not be defined. Instead, pairwise epistasis should be unconditional on the state of other loci in the genome. How do you calculate this?

We assert that pairwise epistasis between two loci should depend on the fitness effect of mutations at those loci where the states of the other loci are determined by mutation–selection balance in a population. In other words, we assert that fitness effects should be measured by the effect on the growth rate of a population. For the three locus system, the fitness of the BC system depends on the frequency of the A = 0 allele and the A = 1 allele in the population. Let *p*_*0*_ stand for the frequency of the A = 0 allele, with *p*_*0*_ *+ p*_*1*_ = *1*. Then

$$\begin{array}{l} w_{00}=p_{0}w_{000}+p_{1}w_{100}\\ w_{01}=p_{0}w_{001}+p_{1}w_{101}\\ w_{10}=p_{0}w_{010}+p_{1}w_{110}\\ w_{11}=p_{0}w_{011}+p_{1}w_{111} \end{array}$$ (4A–4D)

These four values can be used to calculate the epistasis between loci B and C unconditional on the state of A as

$$\varepsilon_{BC}=\text{log}\left(\frac{w_{00}w_{11}}{w_{10}w_{01}}\right)$$ (5)

**Fig 1:**
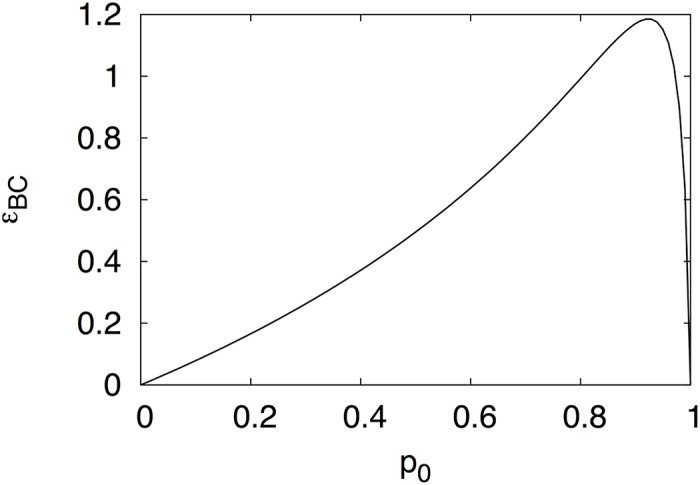
Unconditional epistasis. Pairwise epistasis between loci B and C calculated using Eqs 4 and 5 as a function of locus A allele frequency *p*_*0*_, with fitnesses as in Eq 1.

We plot this quantity in Fig 1 as a function of the frequency of the A = 0 allele *p*_*0*_ and see that it is everywhere positive except for *p*_0_ = 0 or *p*_0_ = 1, which are the conditional epistasis values of Crona (Eqs 2 and 3).

However, given the fitness landscape Eq 1, these extreme values (a population composed purely of one allelic state of A) are impossible. As long as the mutation rate is nonvanishing, there will always be a mixture of both alleles at locus A. Indeed, Table 1 of the supplementary information of [1], which tabulates an evolutionary simulation on that precise landscape, makes that point for us. Crona finds that *p*_0_ ≈ 0.998 in equilibrium, leading to *ε*_*BC*_ ≈ 0.191, which is nonvanishing. Thus, the positive shared entropy between those loci is indeed sufficient to determine nonvanishing pairwise epistasis between them. We also remark that at that frequency *p*_*0*_, the information between B and C is exceedingly small: *I*≈0.0013.

Needless to say, the example discussed here is a fairly contrived one, and we show in Fig 9 of [2] that the correlation between epistasis and information is robust when testing random fitness landscapes. The same argument holds for examples 2 and 3 in the comment [1].

In conclusion, the assertion in [1] that detecting epistasis via shared entropy gives false positives for epistasis is based on a calculation of conditional epistasis, a concept that is ambiguous at best as it depends on the allelic state of all the other loci on the genome and could take on arbitrary values. If epistasis is calculated by averaging over the allelic state of the other loci, then our assertion that correlation (positive information) implies positive pairwise epistasis holds without exception.
